# To transplant or not to transplant during a pandemic?

**DOI:** 10.1590/2175-8239-JBN-2023-E007en

**Published:** 2023-04-03

**Authors:** José Medina Pestana

**Affiliations:** 1 Fundação Oswaldo Ramos, Hospital do Rim, São Paulo, SP, Brazil.

During the early period of the COVID-19 pandemic, most kidney transplant centers hesitated on whether to interrupt transplant procedures, resulting in a significant and universal reduction in the number of kidney transplants^1^. There was a pervasive debate around the risk of transmission to patients and to health care professionals engaged in organ procurement, and on the influence of immunosuppression on the clinical course of COVID-19. Additionally, there was an obvious need to allocate staff and beds in high complexity hospitals to treat a fast-growing number of patients with severe forms of COVID-19. The dilemma was further complicated considering the perspective of overwhelming dialysis units if the number of kidney transplants was reduced drastically, in addition of a significant proportion of patients developing COVID-19 associated acute kidney injury requiring renal replacement therapy.

The study by Teichmann et al.^2^, performed before any vaccine was available, analyzed the incidence, lethality and risk factors associated with SARS-CoV-2 in patients receiving hemodialysis, patients on the waiting list for a kidney transplant, and in kidney transplant recipients. In the study period, patients receiving hemodialysis and recipients of kidney transplant presented a high incidence of COVID-19 and high COVID-19-related lethality. The impact on the patients on the transplant waiting list was less pronounced, most likely because they were younger and had fewer limiting comorbidities.

The reported 9.85% incidence and 14.9% lethality of COVID-19 among kidney transplant recipients is relatively lower compared to other studies in the country, influenced perhaps by the migratory pattern of the disease, clinical surveillance strategies and testing availability^3–5^. While elective living donor kidney transplants were postponed, many transplant centers persisted performing transplants with deceased donor kidneys, depending to the local impact of the pandemic on health care resources, and highly encouraged by frequent videoconferences with experts and transplant physicians promoted by the *Associação Brasileira de Transplant de Órgãos* (ABTO). Therefore, by the end of 2020, the overall reduction in the number of kidney transplants was only 30%, much lower than that observed in other countries^1,6^.

Transplant activity progressively increased with the widespread availability of testing, increased knowledge of the disease and treatment options, availability of vaccines, and the observed reduction in lethality rates in high-risk groups, including the elderly, patients with chronic kidney disease, and recipients of a kidney transplant, further mitigating the burden to dialysis units. After the observation that SARS-CoV-2 could not be transmitted through solid organs, except for the lungs, most centers even began to perform transplantation using organs from individuals who tested positive for SARS-CoV-2 but died for other causes^7^.

Although large-scale vaccination has reduced the need for, and length of, hospitalization and disease lethality in the general population and dialysis patients, such benefit was not immediately observed among kidney transplant recipients. In this group, the humoral and cellular response to vaccination was low, and lethality was sustained in more than twenty-five percent of infected patients, possibly associated with the chronic use of immunosuppressive drugs^4,8^. The situation remained unchanged regardless of virus variant and number or origin of vaccines offered, until the advent of the omicron variant, when the fatality rate among kidney transplant patients was lowered from 25% to 2%^9^.

During the pandemic, many chemical or biological drugs were tested to decrease lethality from COVID-19. However, none achieved clinical meaningful results. Antiviral drugs such as paxlovid are available in the public healthcare system for early treatment, but the extent of their benefit both in the general population and transplant recipients has not yet been established^10,11^. Thus, transplant patients should continue to use face masks in contexts of greater risk, such as when in and around crowds, and be vaccinated whenever recommended.

Finally, initiatives coordinated by the *Sistema National de Transplantes* (SNT) and ABTO have also been implemented to fully restore and even increase the annual number of kidney transplants, with strategies directed to the four pillars of the national transplant system, namely, reporting hospitals, organ procurement organizations, dialysis clinics, and transplant centers.

Kidney transplant is a highly complex and integrated procedure that saves lives. Despite the initial hesitance, in view of the findings reported by Teichmann et al.^2^, the decision to maintain and foment the transplant activity, considering the local environment during the evolving phases of the COVID-19 pandemic, in retrospect, appears to have been the right decision.
